# Supplementary material for: Vaccination strategies impact the probability of outbreak extinction: A case study of COVID-19 transmission
Source: Heliyon. 2024 Mar 15;10(6):e28042. doi: 10.1016/j.heliyon.2024.e28042 (PMC10958689; doi:10.1016/j.heliyon.2024.e28042)
Supplement: Multimedia component 1 [file mmc1.pdf]

Supplementary Material:

Vaccination strategies impact the probability of outbreak extinction: a case study of COVID-19 transmission

Natcha C. Jitsuk[1,2], Sudarat Chadsuthi[2,3], and Charin Modchang[1,2,4,5*]

[1] Biophysics Group, Department of Physics, Faculty of Science, Mahidol University, Bangkok 10400, Thailand

[2] Center for Disease Modeling, Faculty of Science, Mahidol University, Bangkok, 10400, Thailand

[3] Department of Physics, Research Center for Academic Excellence in Applied Physics, Faculty of Science, Naresuan University, Phitsanulok 65000, Thailand

[4] Centre of Excellence in Mathematics, MHESI, Bangkok 10400, Thailand

[5] Thailand Center of Excellence in Physics, Ministry of Higher Education, Science, Research and Innovation, 328 Si Ayutthaya Road, Bangkok 10400, Thailand

* Corresponding author: Charin Modchang

Biophysics Group, Department of Physics, Faculty of Science, Mahidol University, Bangkok 10400, Thailand

Email : charin.mod@mahidol.edu

1. Disease transmission model

The following ordinary differential equations describe the rates of change of population in age group *i*:

$$\frac{dS_i}{dt} = -\sum_{j=1}^{16} \frac{\beta_{ij}}{N}\left(I_j + q_A A_j + (1 - e_I)\left(I_{P_j} + q_A A_{P_j}\right)\right) S_i,$$

$$\frac{dL_i}{dt} = \sum_{j=1}^{16} \frac{\beta_{ij}}{N}(I_j + q_A A_j + (1 - e_I)\left(I_{P_j} + q_A A_{P_j}\right))S_i - \sigma L_i,$$

$$\frac{dI_i}{dt} = \sigma(1 - f_A)L_i - \gamma I_i,$$

$$\frac{dA_i}{dt} = \sigma f_A L_i - \gamma A_i,$$

$$\frac{dR_i}{dt} = \gamma((1 - f_D)I_i + A_i),$$

$$\frac{dD_i}{dt} = \gamma f_D I_i,$$

$$\frac{dS_{P_i}}{dt} = -\sum_{j=1}^{16} \frac{\beta_{ij}}{N}(I_j + q_A A_j + (1 - e_I)\left(I_{P_j} + q_A A_{P_j}\right))S_{P_i},$$

$$\frac{dL_{P_i}}{dt} = -\sum_{j=1}^{16} \frac{\beta_{ij}}{N}(I_j + q_A A_j + (1 - e_I)\left(I_{P_j} + q_A A_{P_j}\right))S_{P_i} - \sigma L_{P_i},$$

$$\frac{dI_{P_i}}{dt} = \sigma(1 - f_{AP})L_{P_i} - \gamma I_{P_i},$$

$$\frac{dA_{P_i}}{dt} = \sigma f_{AP} L_{P_i} - \gamma A_{P_i},$$

$$\frac{dR_{P_i}}{dt} = \gamma\left((1 - f_{DP})I_{P_i} + A_{P_i}\right),$$

$$\frac{dD_{P_i}}{dt} = \gamma f_{DP} I_{P_i}.$$

The abovementioned differential equations are advantageous for considering the number of events that occur in each time step. **Table S1** displays the examples of the number of events that occur in each time step.

Table S1: The example of the number of events that occur in each time step.

**Event**	**Number of events in each time step, τ**
S_i is infected from I_j	Poisson($\tau \times \frac{\beta_{ij} S_i I_j}{N}$) ; j = 1:16
S_i is infected from I_{Pj}	Poisson($\tau \times \frac{(1-e_I)\beta_{ij} S_i I_{p,j}}{N}$) ; j = 1:16
S_i is infected from A_j	Poisson($\tau \times \frac{q_A \beta_{ij} S_i A_j}{N}$) ; j = 1:16
S_i is infected from A_{Pj}	Poisson($\tau \times \frac{(1-e_I) q_A \beta_{ij} S_i A_{p,j}}{N}$) ; j = 1:16

Table S2: Variables and their definitions

**Variables**	**Definition**
S	Unvaccinated susceptible individual
L	Unvaccinated latently infected individual
I	Unvaccinated symptomatic infectious individual
A	Unvaccinated asymptomatic infectious individual
R	Unvaccinated recovered individual
D	Unvaccinated death
S_P	Partially immunized susceptible individual
L_P	Partially immunized latently infected individual
I_P	Partially immunized symptomatic infectious individual
A_P	Partially immunized asymptomatic infectious individual
R_P	Partially immunized recovered individual

D_P	Partially immunized death
V	Perfectly immunized individual

Table S3: Vaccine effectiveness parameters

Parameters	Definition	High vaccine effectiveness scenario (References)	Low vaccine effectiveness scenario (References)
e_I	Effectiveness against transmission	0.68 ([1])	0.032 ([2])
e_S	Effectiveness against infection	0.95 ([3])	0.55 ([4])
e_D	Effectiveness against disease	0.97 ([3, 5, 6])	0.49 ([7])
e_{DE}	Effectiveness against death	0.96 ([3, 8])	0.95 ([9])

2. Estimating the fraction of vaccinated asymptomatic infection

According to the definition of the vaccine efficacy against disease,

$$e_D = \frac{\text{disease in unvaccinated group - disease in vaccinated group}}{\text{disease in unvaccinated group}},$$

$$e_D = \frac{\sigma(1 - f_A) - \sigma(1 - e_S)(1 - f_{AP})}{\sigma(1 - f_A)},$$

where

σ = Transition rate of individuals in the latent compartment to the infectious compartment,

f_A = Proportion of asymptomatic individuals,

f_{AP} = Proportion of asymptomatic vaccinated individuals,

e_S = Vaccine effectiveness against infection,

e_I = Vaccine effectiveness against transmission,

e_D = Vaccine effectiveness against symptomatic disease,

which can be simplified to

$$e_D = 1 - \frac{(1 - e_S)(1 - f_{AP})}{(1 - f_A)}.$$

Then, f_{AP} is calculated from the following equation:

$$f_{AP} = 1 - \frac{(1 - f_A)(1 - e_D)}{(1 - e_S)}$$

3. Estimating the proportion of symptomatic infected individuals who eventually die

We estimated the fraction of symptomatic infected individuals who eventually die using the definition of the infection fatality ratio (IFR) which represents the proportion of deaths among all infected individuals, including all asymptomatic and undiagnosed infected individuals:

$$\text{IFR} = \frac{\text{No. of deaths}}{\text{No. of infected individuals}},$$

$$\text{IFR} = \frac{N_D}{N_I},$$

where $N_I = (1 - f_A)N_I + f_A N_I$ and $N_D = f_D(1 - f_A)N_I$,

f_A = Proportion of asymptomatic individuals,

f_D = Proportion of deaths,

N_I = Number of infected individuals,

N_D = Number of deaths,

We plugged them into the equation

$$\text{IFR} = \frac{f_D(1-f_A)N_I}{(1-f_A)N_I + f_A N_I},$$

$$\text{IFR} = f_D(1 - f_A).$$

Thus, $$f_D = \frac{\text{IFR}}{1-f_A}.$$

4. Estimating the proportion of symptomatic breakthrough-infected individuals who eventually die

Vaccine effectiveness is measured by calculating the risk of disease among vaccinated and unvaccinated persons and determining the percentage reduction in risk of disease among vaccinated persons relative to unvaccinated persons. The vaccine effectiveness against death is given by

$$e_{ED} = \frac{\frac{D}{1-a} - \frac{D_P}{a}}{\frac{D}{1-a}} = 1 - \frac{D_P(1-a)}{aD}.$$

where

e_{DE} = vaccine effectiveness against death,

D = number of deaths in unvaccinated population,

D_P = number of deaths in vaccination population,

f_A = Proportion of asymptomatic individuals,

f_{AP} = Proportion of asymptomatic vaccinated individuals,

f_D = Proportion of deaths,

e_S = Vaccine effectiveness against infection,

e_I = Vaccine effectiveness against transmission,

e_D = Vaccine effectiveness against symptomatic disease,

q_A = Asymptomatic Infectiousness.

From the model, we have $S = 1 - a$ where a is the vaccine coverage (fraction of vaccinated population), $S_P = (1 - e_S)a$, and $S_V = e_S a$. Let $f_{DP} = Cf_D$, where C is a scaling parameter. Then,

$D_P = (1 - e_S)a\lambda\sigma(1 - f_{AP})\gamma f_D C,$

$D = (1 - a)\lambda\sigma(1 - f_A)\gamma f_D.$

We substituted D_P and D into

$$e_{ED} = 1 - \frac{D_P(1-a)}{aD}.$$

We then got

$$e_{ED} = 1 - \frac{(1 - e_S)a\lambda\sigma(1 - f_{AP})\gamma f_D C(1 - a)}{(1 - a)\lambda\sigma(1 - f_A)\gamma f_D a},$$

$$e_{ED} = 1 - \frac{(1 - e_S)(1 - f_{AP})C}{(1 - f_A)}.$$

To simplify this equation, we employed f_A from the following equation

$$f_{AP} = 1 - \frac{(1 - f_A)(1 - e_D)}{(1 - e_S)}.$$

And rearrange

$$(1 - f_{AP}) = \frac{(1 - f_A)(1 - e_D)}{(1 - e_S)}.$$

Then,

$$e_{ED} = 1 - \frac{(1 - e_S)(1 - f_A)(1 - e_D)C}{(1 - f_A)(1 - e_S)},$$

$$e_{ED} = 1 - (1 - e_D)C,$$

$$1 - e_{ED} = (1 - e_D)C.$$

Therefore,

$$C = \frac{(1 - e_{ED})}{(1 - e_D)}.$$

Finally, we got the relation of f_{DP} and f_D

$$f_{DP} = \frac{(1 - e_{ED})}{(1 - e_D)} f_D.$$

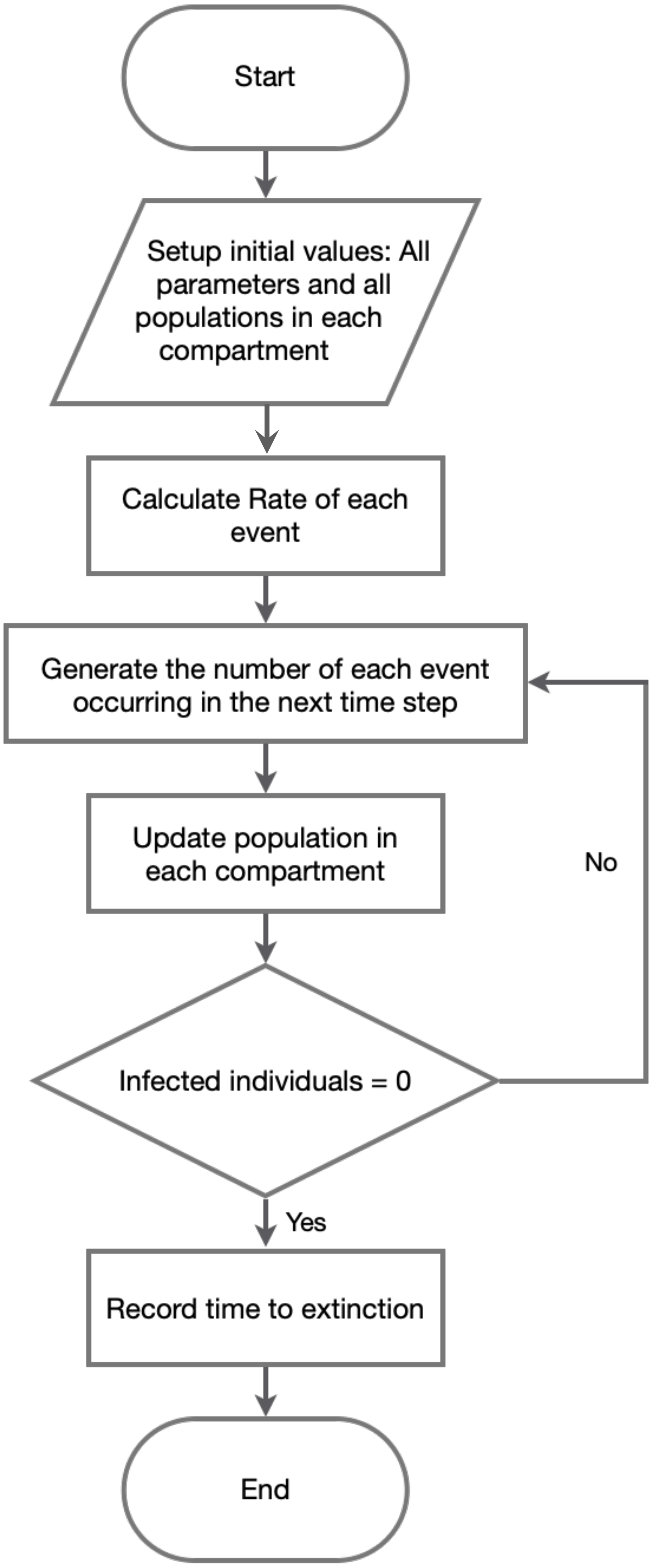

Fig. S1: Model algorithm flowchart.

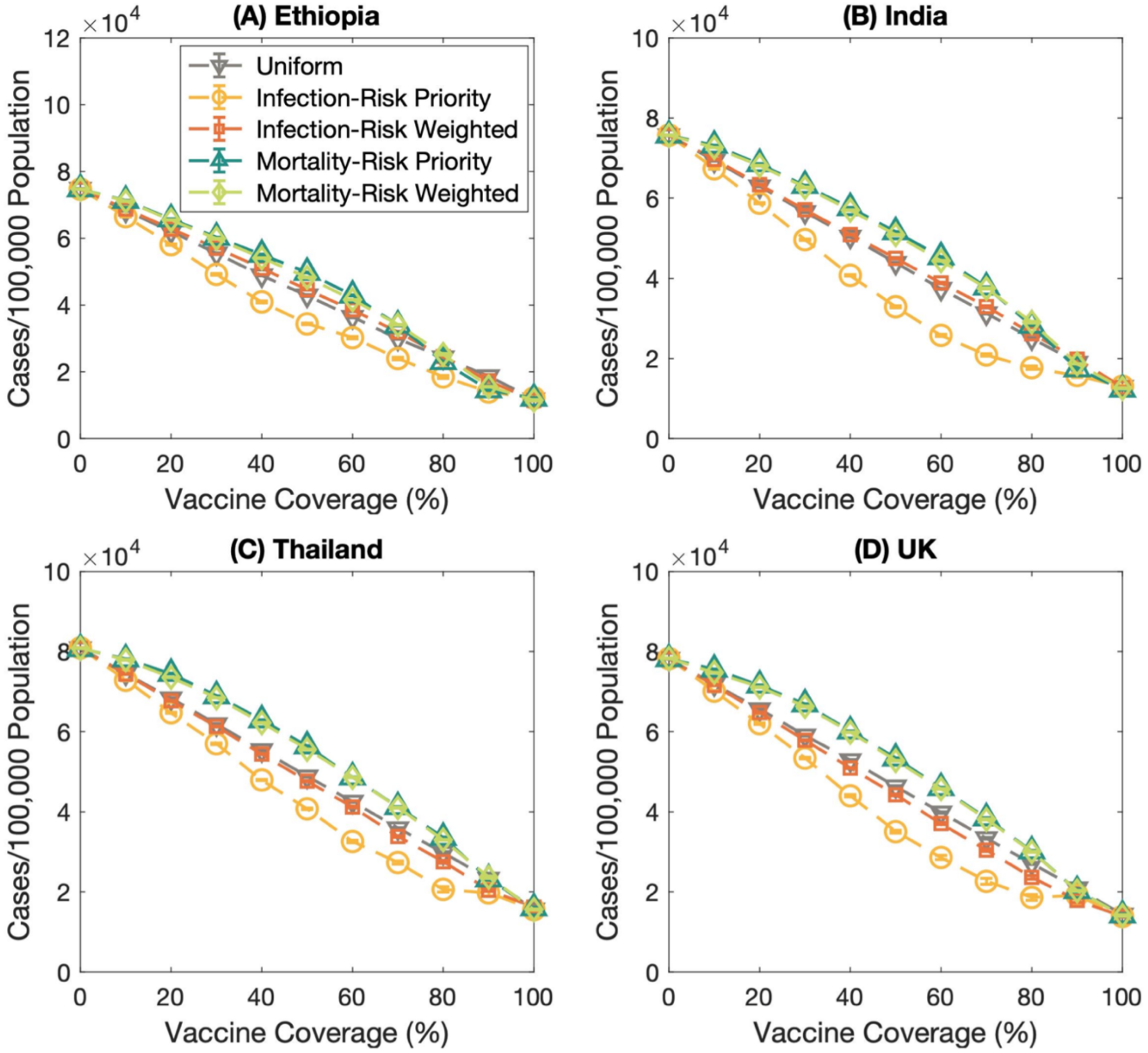

Fig. S2 The number of cases per 100,000 population of various countries; **(A)** Ethiopia, **(B)** India, **(C)** Thailand, and **(D)** the United Kingdom. The dashed lines with the downward-pointing triangle, the circle, the square, and the upward-pointing triangle, and the diamond marks represent the number of cases per 100,000 population of the uniform, infection-risk priority, infection-risk weighted, mortality-risk priority, and mortality-risk weighted vaccination strategies, respectively. Error bars show the standard error.

Table S4: Vaccine effectiveness against Omicron variants. Data were obtained from [1, 4, 7, 10, 11].

month	e_S	e_I	e_D	e_{DE}
1	0.55	0.032	0.49	0.95
2	0.16	0.032	0.30	0.93
3	0.098	0.032	0.15	0.83
**booster**				
1	0.55	0.077	0.67	0.95

5. Sensitivity Analysis

5.1 The effect of vaccination strategies on the outbreak extinction probability

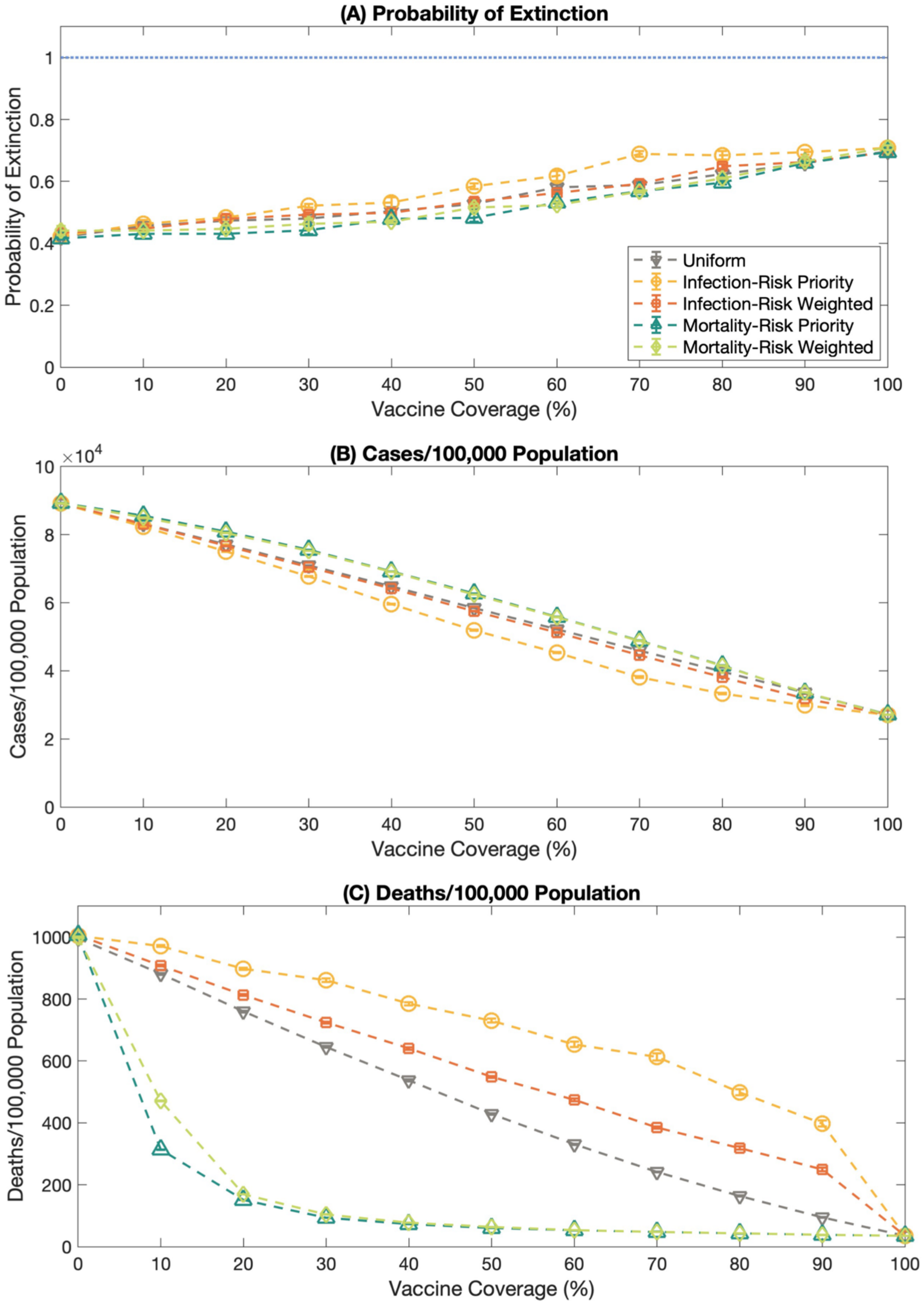

Fig. S3 The impact of various vaccination strategies on the extinction probability, the infection rate, and the mortality rate. $R_0 = 3.5$.

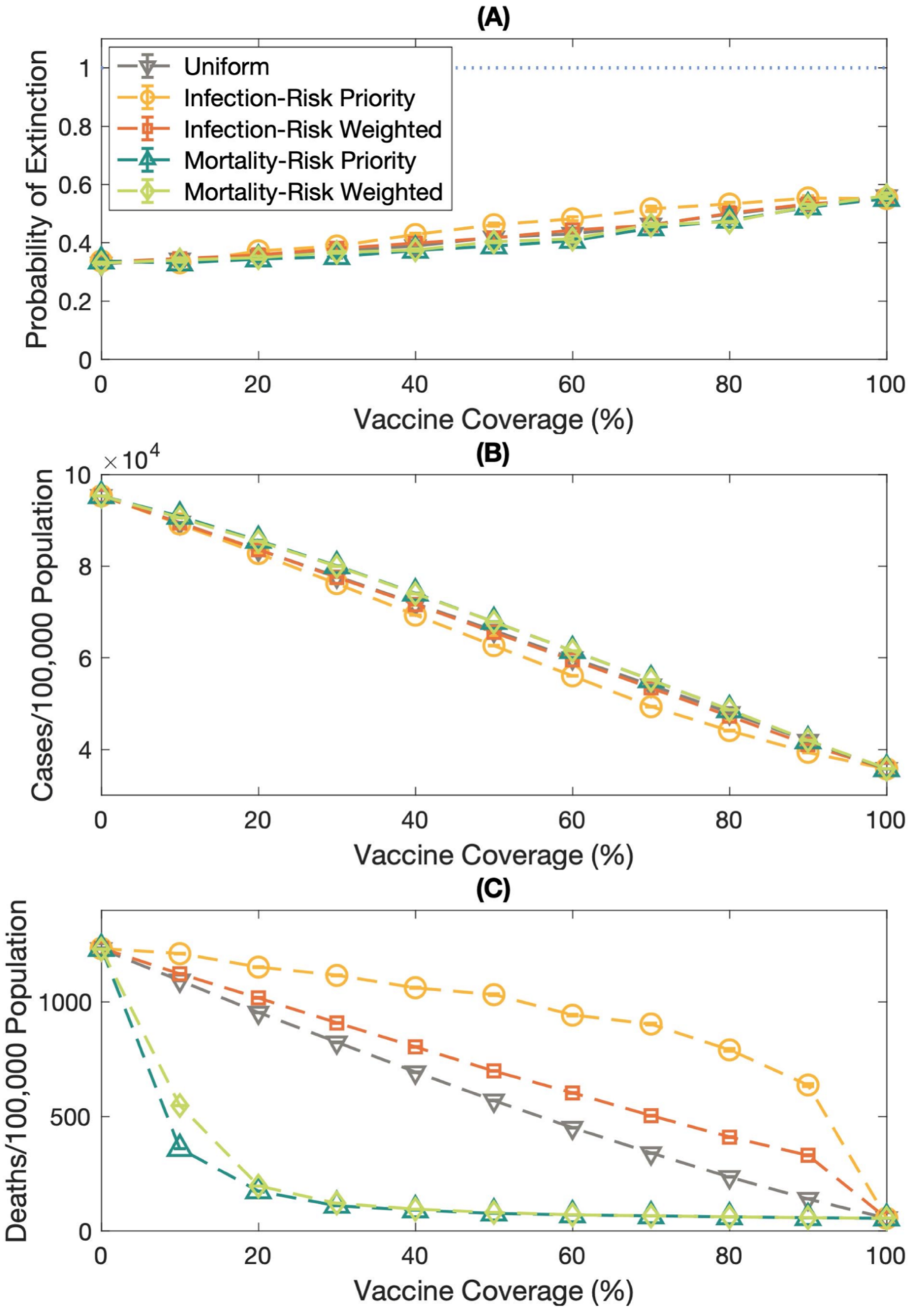

Fig. S4 The impact of various vaccination strategies on the extinction probability, the infection rate, and the mortality rate. $R_0 = 5.0$.

5.2 The effect of waning vaccine effectiveness on the outbreak extinction probability

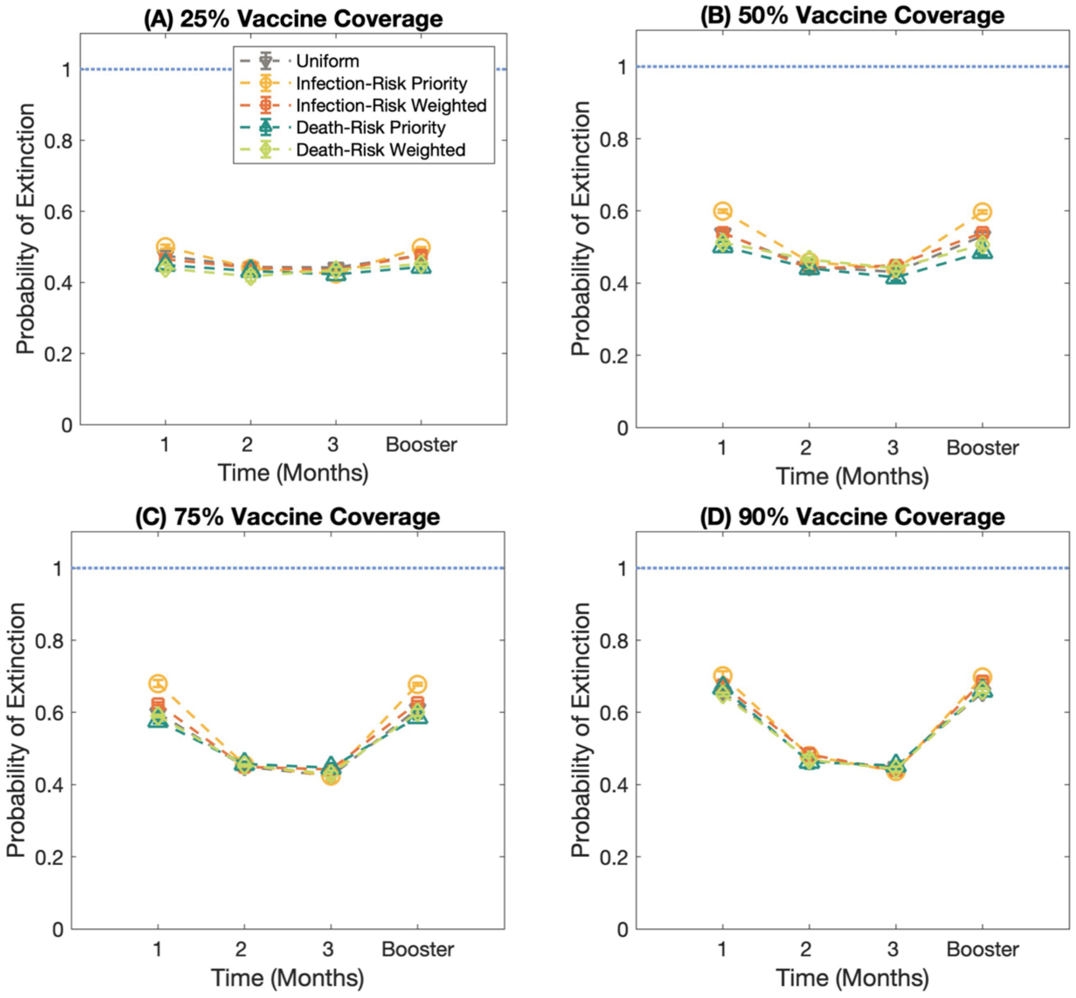

Fig. S5 The effect of waning vaccine effectiveness against the Omicron variant (B.1.1.529) on the probability of outbreak extinction. **(A) – (D)** display the extinction probabilities under the different vaccine coverages; 25%, 50%, 75%, and 90% of the total population, respectively. Each line represents the extinction probability of different vaccination strategies. $R_0 = 3.5$.

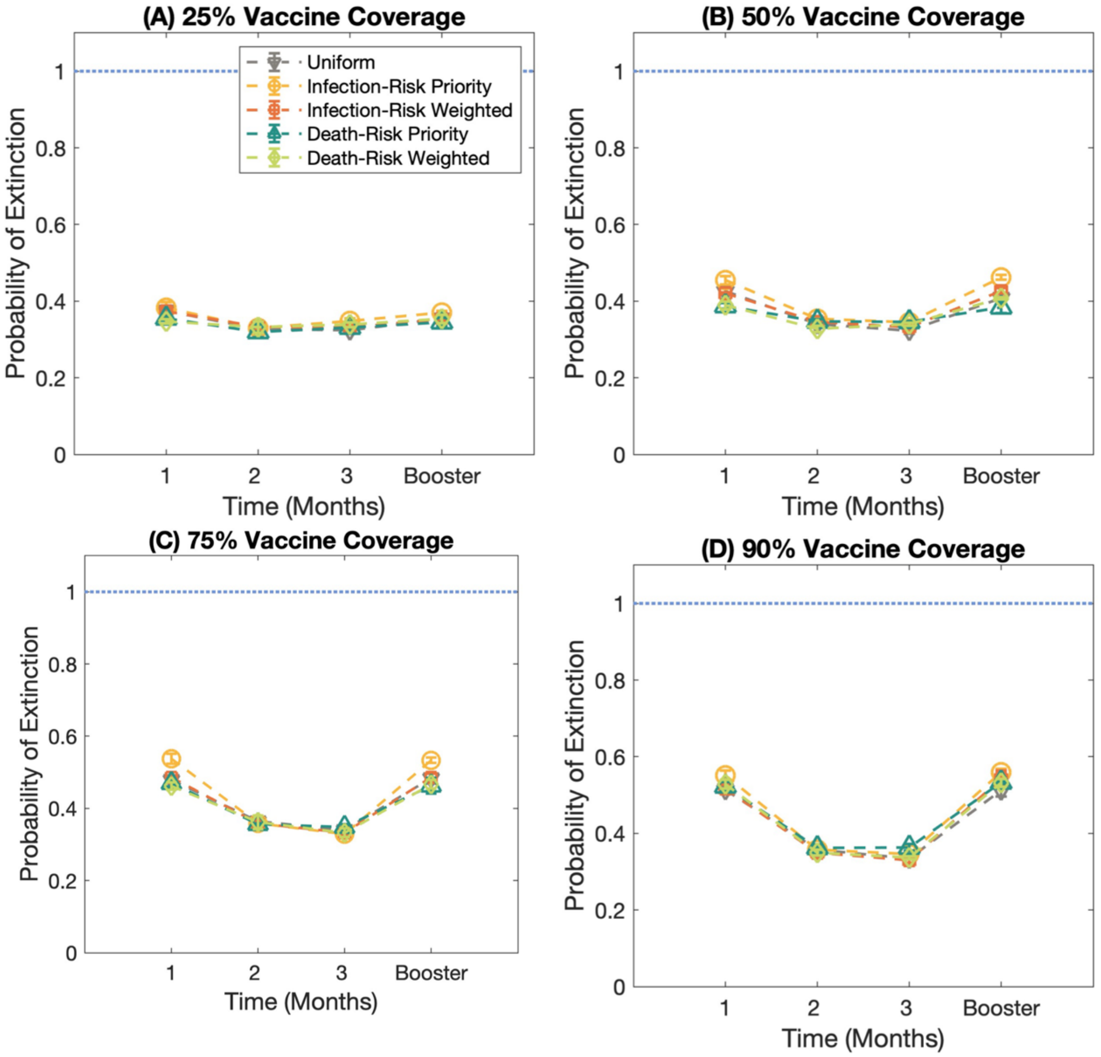

Fig. S6 The effect of waning vaccine effectiveness against the Omicron variant (B.1.1.529) on the probability of outbreak extinction. **(A) – (D)** display the extinction probabilities under the different vaccine coverages; 25%, 50%, 75%, and 90% of the total population, respectively. Each line represents the extinction probability of different vaccination strategies. $R_0 = 5.0$.

5.3 The effect of waning vaccine effectiveness on the cases

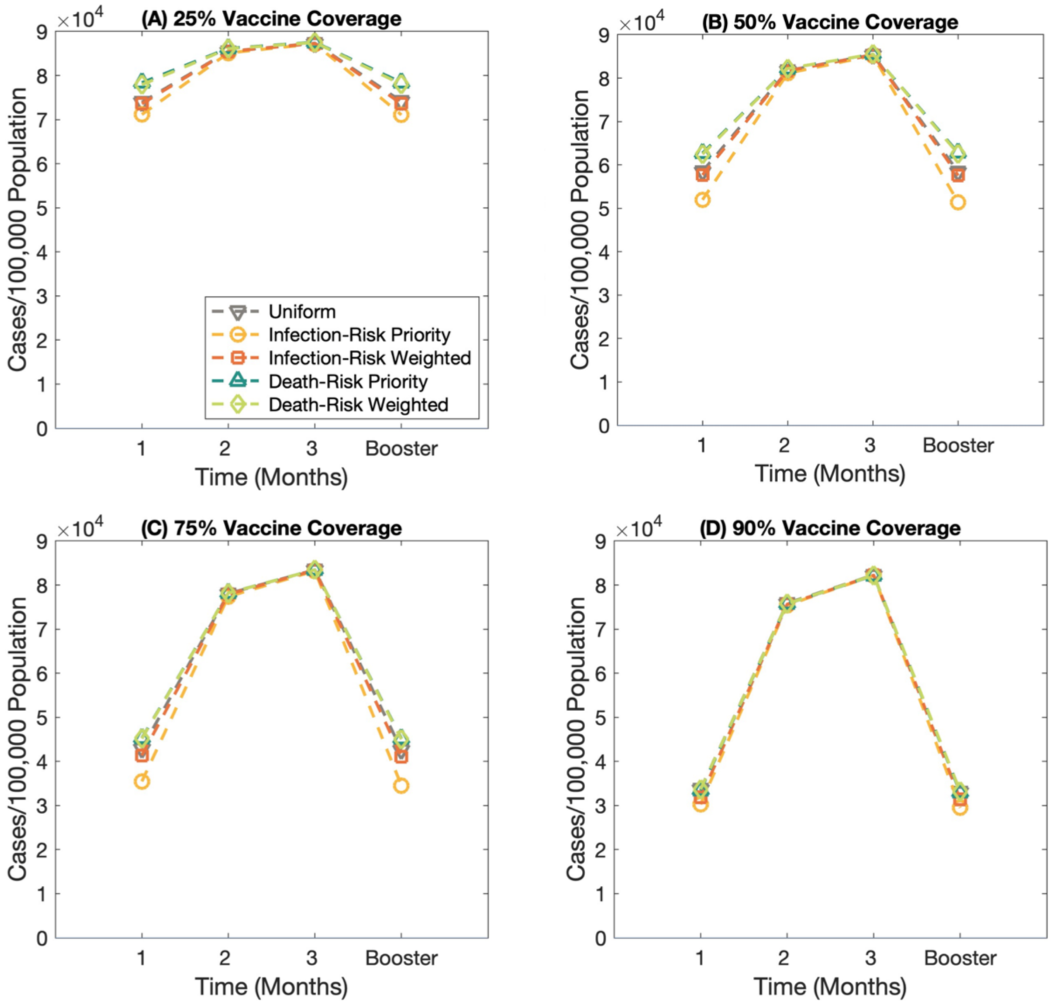

Fig. S7 The effect of waning vaccine effectiveness against the Omicron variant (B.1.1.529) on the cases per 100,000 population. **(A) – (D)** display the cases per 100,000 population under the different vaccine coverages: 25%, 50%, 75%, and 90% of the total population, respectively. Each line represents the cases per 100,000 population of different vaccination strategies. $R_0 = 3.5$.

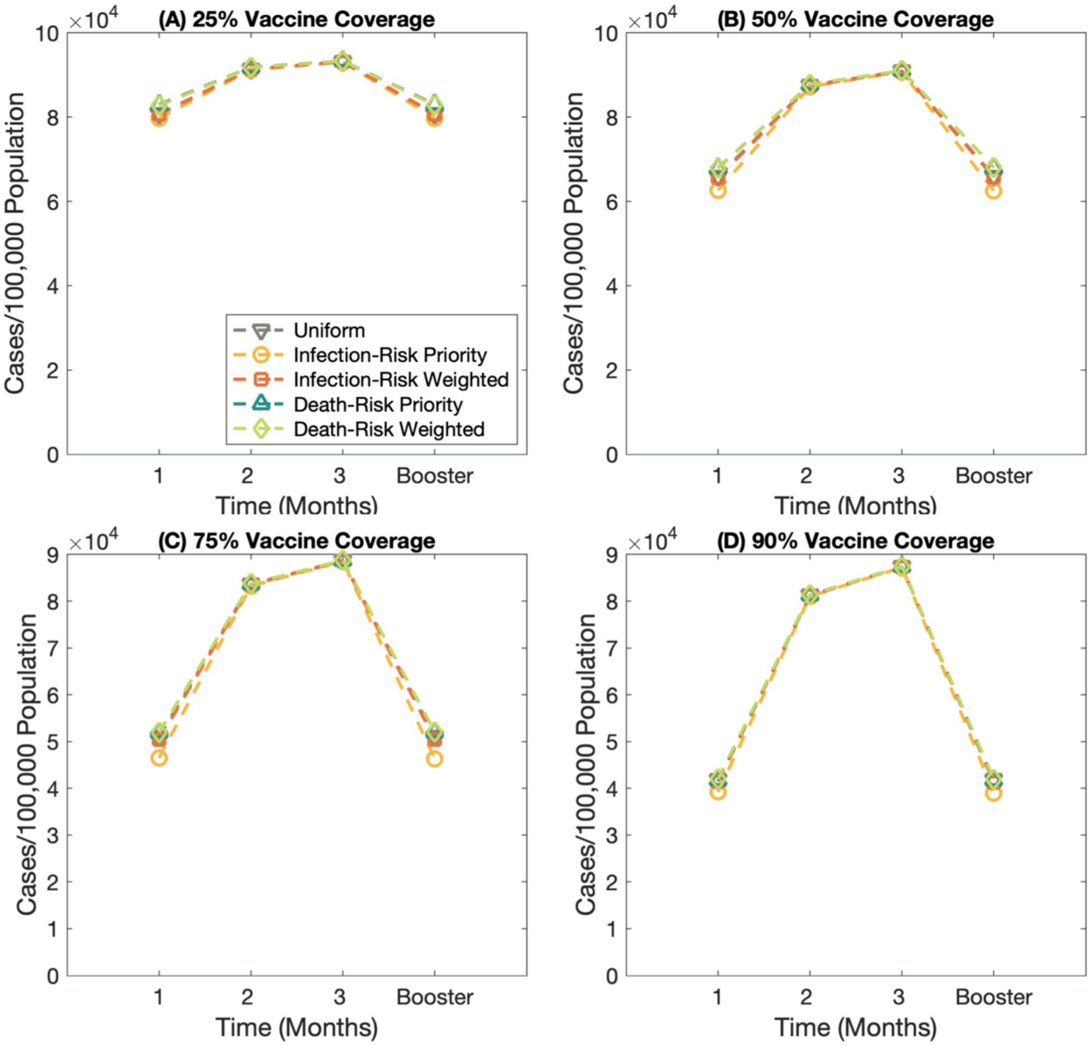

Fig. S8 The effect of waning vaccine effectiveness against the Omicron variant (B.1.1.529) on the cases per 100,000 population. **(A) – (D)** display the cases per 100,000 population under the different vaccine coverages: 25%, 50%, 75%, and 90% of the total population, respectively. Each line represents the cases per 100,000 population of different vaccination strategies. $R_0 = 5.0$.

5.3 The effect of waning vaccine effectiveness on the deaths

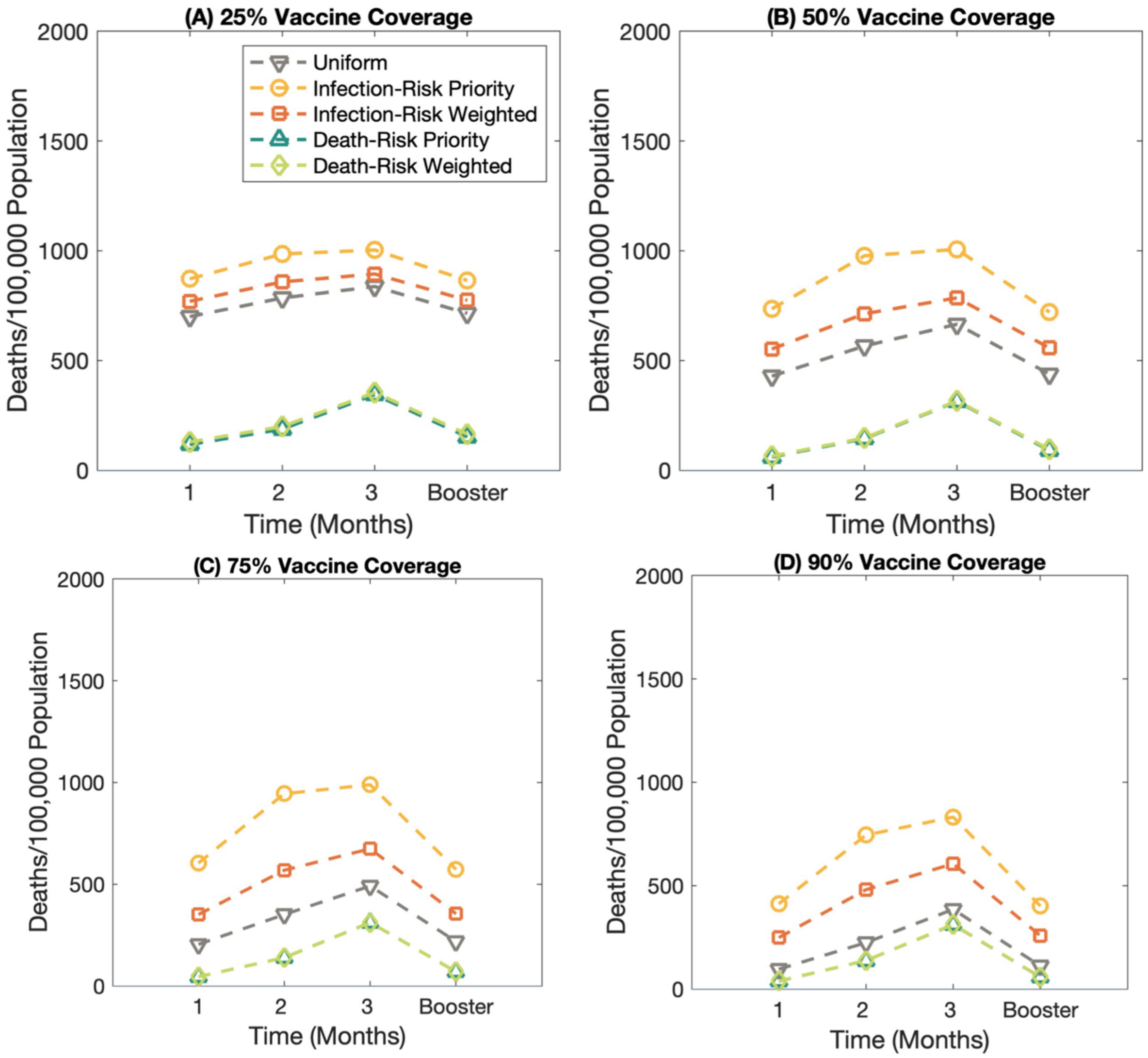

Fig. S9 The effect of waning vaccine effectiveness against the Omicron variant (B.1.1.529) on the deaths per 100,000 population. **(A) – (D)** display the deaths per 100,000 population under the different vaccine coverages: 25%, 50%, 75%, and 90% of the total population, respectively. Each line represents the deaths per 100,000 population of different vaccination strategies. $R_0 =$ 3.5.

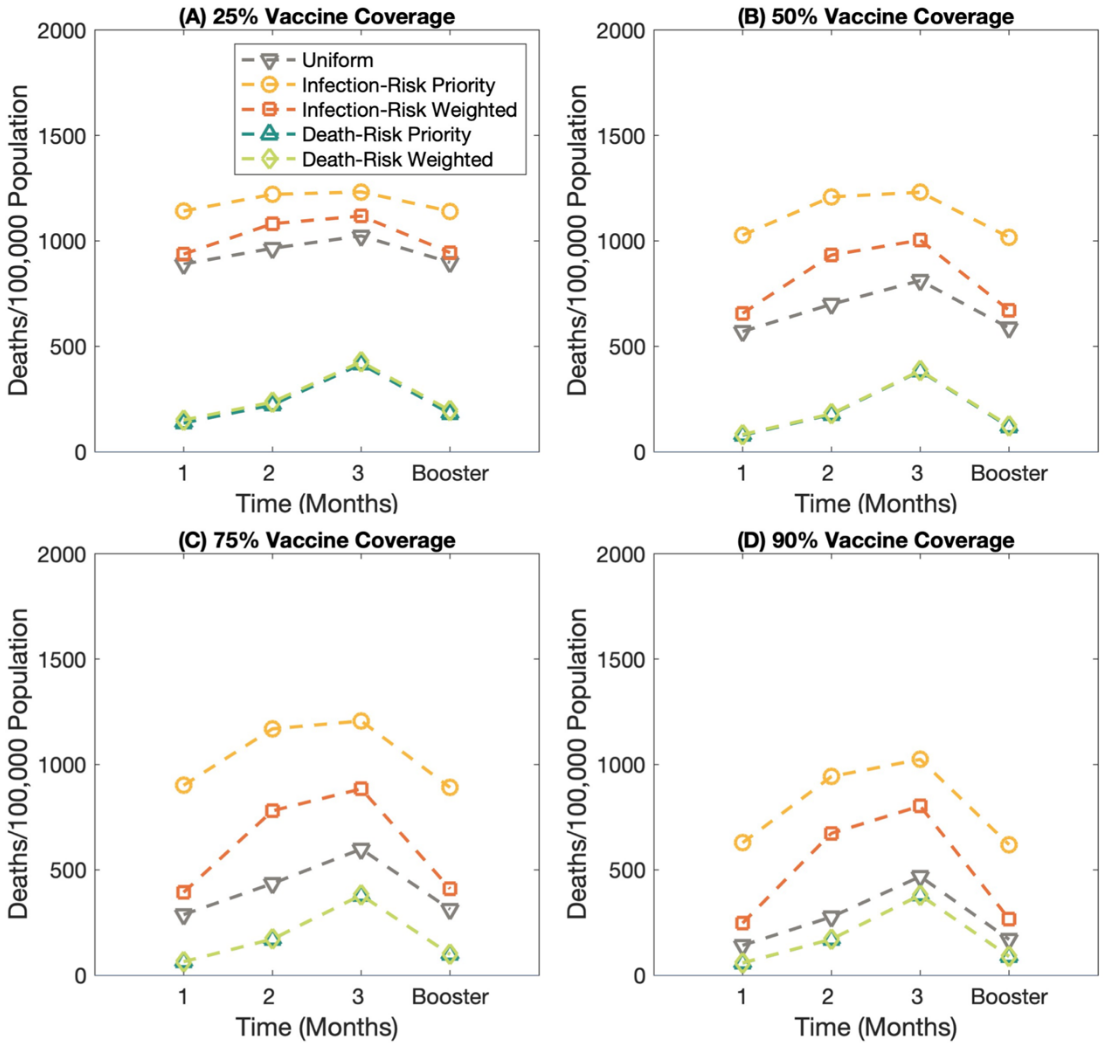

Fig. S10 The effect of waning vaccine effectiveness against the Omicron variant (B.1.1.529) on the deaths per 100,000 population. **(A) – (D)** display the deaths per 100,000 population under the different vaccine coverages: 25%, 50%, 75%, and 90% of the total population, respectively. Each line represents the deaths per 100,000 population of different vaccination strategies. $R_0 = 5.0$.

References

1. Eyre, D.W., et al., *Effect of Covid-19 Vaccination on Transmission of Alpha and Delta Variants.* New England Journal of Medicine, 2022.
2. Gardner, B.J. and A.M. Kilpatrick, *Estimates of reduced vaccine effectiveness against hospitalization, infection, transmission and symptomatic disease of a new SARS-CoV-2 variant, Omicron (B. 1.1. 529), using neutralizing antibody titers.* MedRxiv, 2021.
3. Haas, E.J., et al., *Impact and effectiveness of mRNA BNT162b2 vaccine against SARS-CoV-2 infections and COVID-19 cases, hospitalisations, and deaths following a nationwide vaccination campaign in Israel: an observational study using national surveillance data.* The Lancet, 2021. **397**(10287): p. 1819-1829.
4. Hansen, C.H., et al., *Vaccine effectiveness against SARS-CoV-2 infection with the Omicron or Delta variants following a two-dose or booster BNT162b2 or mRNA-1273 vaccination series: A Danish cohort study.* medRxiv, 2021.
5. Abu-Raddad, L.J., H. Chemaitelly, and A.A. Butt, *Effectiveness of the BNT162b2 Covid-19 Vaccine against the B. 1.1. 7 and B. 1.351 Variants.* New England Journal of Medicine, 2021.
6. Moghadas, S.M., et al., *Evaluation of COVID-19 vaccination strategies with a delayed second dose.* PLoS biology, 2021. **19**(4): p. e3001211.
7. Andrews, N., et al., *Covid-19 vaccine effectiveness against the Omicron (B. 1.1. 529) variant.* New England Journal of Medicine, 2022. **386**(16): p. 1532-1546.
8. Nasreen, S., et al., *Effectiveness of COVID-19 vaccines against symptomatic SARS-CoV-2 infection and severe outcomes with variants of concern in Ontario.* Nature Microbiology, 2022. **7**(3): p. 379-385.
9. Chemaitelly, H., et al., *Duration of protection of BNT162b2 and mRNA-1273 COVID-19 vaccines against symptomatic SARS-CoV-2 Omicron infection in Qatar.* medRxiv, 2022.
10. Agency, U.H.S., *COVID-19 vaccine surveillance report Week 12*. 2022.
11. Ferguson, N., et al., *Report 49: Growth, population distribution and immune escape of Omicron in England.* Imperial College London, 2021. **16**.
